# Is the Training Intensity in Phase Two Cardiovascular Rehabilitation Different in Telehealth versus Outpatient Rehabilitation?

**DOI:** 10.3390/jcm10184069

**Published:** 2021-09-09

**Authors:** Ladislav Batalik, Garyfallia Pepera, Jannis Papathanasiou, Sebastian Rutkowski, David Líška, Katerina Batalikova, Martin Hartman, Marián Felšőci, Filip Dosbaba

**Affiliations:** 1 Department of Rehabilitation, University Hospital Brno, 62500 Brno, Czech Republic; batalikova.katerina@fnbrno.cz (K.B.); hartman.martin@fnbrno.cz (M.H.); dosbaba.filip@fnbrno.cz (F.D.); 2 Department of Public Health, Faculty of Medicine, Masaryk University, 62500 Brno, Czech Republic; 3 Physiotherapy Department, Faculty of Health Sciences, University of Thessaly, 35100 Lamia, Greece; gpepera@uth.gr; 4 Department of Medical Imaging, Allergology & Physiotherapy, Faculty of Dental Medicine, Medical University of Plovdiv, 4002 Plovdiv, Bulgaria; giannipap@yahoo.co.uk; 5 Department of Kinesitherapy, Faculty of Public Health “Prof. Dr. Tzecomir Vodenicharov, Ph.D”, Medical University of Sofia, 1431 Sofia, Bulgaria; 6 Faculty of Physical Education and Physiotherapy, Opole University of Technology, 45-758 Opole, Poland; s.rutkowski@po.edu.pl; 7 Faculty of Arts, Department of Physical Education and Sports, Matej Bel University, 97401 Banská Bystrica, Slovakia; david.liska27@gmail.com; 8 Department of Internal Medicine and Cardiology, University Hospital Brno, 62500 Brno, Czech Republic; felsoci.marian@fnbrno.cz; 9 Department of Internal Medicine and Cardiology, Faculty of Medicine, Masaryk University, 62500 Brno, Czech Republic

**Keywords:** cardiovascular rehabilitation, telehealth, physical exercise, coronary artery disease, heart rate disease, telerehabilitation, outpatient rehabilitation

## Abstract

Telehealth cardiac rehabilitation (CR) is a feasible and effective alternative to conventional outpatient CR. Present evidence is limited on the comparison of exercise intensity adherence in telehealth and outpatient CR. The purpose of the study was to evaluate and compare training intensity adherence through 12-week phase II CR in telehealth and outpatient CR. A sample of 56 patients with coronary artery disease (CAD) with a mean age of 56.7 ± 7.1 entering comprehensive secondary prevention phase II was randomized into telehealth CR (*n* = 28) and control outpatient CR (*n* = 28) groups. The primary outcome was a comparison of training intensity adherence in both CR models and heart rate (HR) response from individual CR sessions, expressed by the HR reserve percentage. As a result, the parameter HR reserve percentage as the total average of the training intensity during the telehealth intervention and the outpatient CR did not differ statistically (*p* = 0.63). There was no death case, and all severe adverse cases required medical admission throughout an exercise training session in study subjects in both groups. This research evidence demonstrated that the telehealth CR model is similar in training intensities to the conventional outpatient CR in CAD patients with low to moderate cardiovascular risk.

## 1. Introduction

Coronary artery disease (CAD) is a major global public health problem that requires efforts to develop sustainable approaches to treatment and prevention [1]. Training interventions have been recognized as an essential part of treatment and comprehensive cardiac rehabilitation (CR) in a wide range of patients with heart disease. [2,3]. To help patients with CAD, phase I rehabilitation programs are implemented in the hospital at the time of a cardiac event or procedure, while phase II programs are implemented in the outpatient care setting and usually last 3 months and a maximum of 36 sessions [4]. The standardized model of CR includes risk factor management, structured physical training, patient education, and health and psychosocial behavioral counseling [5]. The implication of exercise-based training interventions is well known, particularly concerning improved cardiorespiratory fitness [6], morbidity, and mortality [7]. Successful completion of the phase II CR should result in significant improvements in physical capacity, quality of life for the patient, and reinforcement of the need for lifestyle changes [8]. It has also been shown that the effectiveness of CR programs depends on several factors, e.g., program delivery (dose and content) and patient motivation [9]. Furthermore, evidence suggests that the outcomes achieved in phase II CR influence the effects of the CR maintenance programs (referred to as phase III or IV depending on the country), which are designed to promote adherence to the healthy lifestyle changes achieved in previous phases, particularly physical exercise [10]. Nevertheless, despite strong recommendations (Class I, Level A), CR implementation is low globally [11,12]. Participation in conventional phase II CR is approximately 40% for rehabilitation services providers in Europe, 30% in the US, and only 23% in middle-income countries [13,14]. Therefore, the emphasis on improving CR implementation by integrating different strategies and alternatives is strongly supported by international societies of CR and prevention [15,16].

As alternatives to in-hospital interventions, telehealth approaches in CR have recently been extensively researched and developed to examine the feasibility, safety, and the effect of the intervention [17,18]. Telehealth covers the delivery of various healthcare services to patients via telecommunications technology, including (1) telemedicine and (2) telehealthcare [19]. Telerehabilitation, which is a part of telemedicine, was defined in 2002 as a set of rehabilitation services for individuals with disabilities provided “remotely” via telecommunications systems and the internet. Systematic reviews suggest that telerehabilitation has become more widely adopted for cardiac patients due to the need to provide equal access to rehabilitation for communities with barriers to traditional models of care [20]. Moreover, current systematic reviews have noted on 7283 CAD participants that telehealth interventions may be an optimal alternative for conventional outpatient CR and recommend subsequent implementation into clinical practice [21], particularly in the current period affected by the global SARS-CoV-2 pandemic where restrictions have highlighted the need to provide CR in a remote state. Consequently, a significant proportion of hospitals and CR providers have been forced to re-organize their activities or reduce services [22,23]; the call for action has significantly increased [24].

However, implementation of telehealth CR services in clinical medicine avoids specific concerns, including legal clarity over intervention responsibilities, costs, and training adherence as well as the issue of compliance with training exercise intensity in the home environment [25]. Therefore, the research question of our study was: Is the training intensity in telehealth versus outpatient phase II CR different? We assumed that the intensity of training recorded in the remote telehealth CR group would be similar to conventional outpatient CR in CAD patients with low to moderate cardiovascular risk.

The findings of this study could address essential concerns regarding the issue that participants in home-based or remote-led CR are achieving satisfactory training adherence. Ultimately, maintaining training intensity is essential to achieving the expected effects of training intervention [26].

## 2. Materials and Methods

### 2.1. Research Design

The present study was a prospective, single-center, two-arm randomized, controlled trial performed from August 2018 to January 2020 at University Hospital Brno, Department of Rehabilitation, Cardiovascular Outpatient Rehabilitation Center. The trial was carried out in compliance with the Helsinki Declaration of Ethical Principles for Medical Research, in which human subjects participate, and was approved by the Ethics Committee of the University Hospital Brno, Czech Republic (approval code NIG2016.5.31). Patients signed an informed consent form before entering the study. According to the World Health Organization recommendations, the study was prospectively registered in the Register of Clinical Trials with International Operations under registration number ACTRN12618001170213. The study inclines to CONSORT guidelines of reporting trials.

### 2.2. Sample

**Figure 1:**
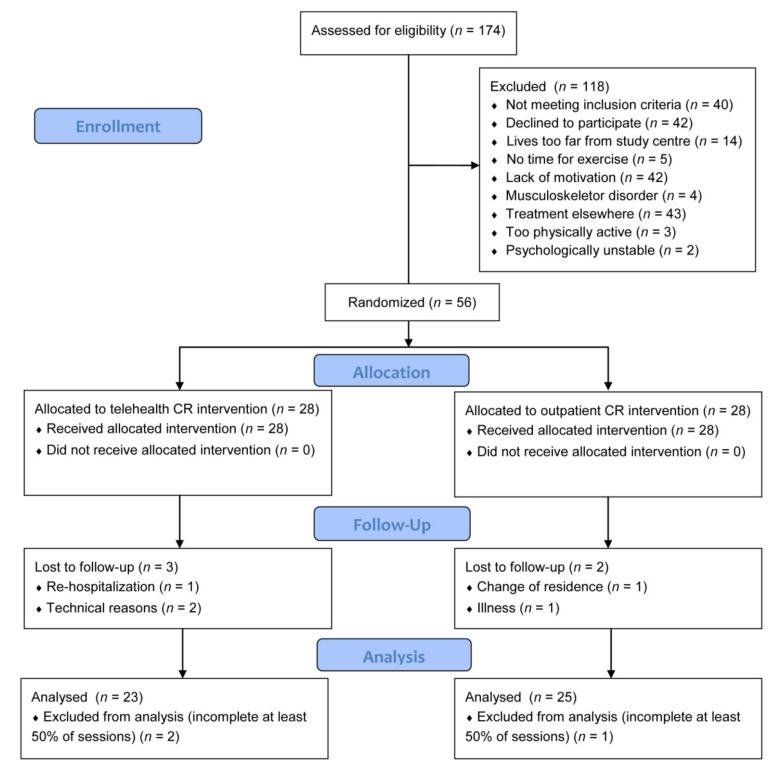
Study flow diagram.

Fifty-six (56) eligible CAD participants (46 men and 10 women) were recruited to participate in 12-week training intervention phase II CR. (Figure 1) A 1:1 randomization followed initial examinations, and patients were allocated to the experimental telehealth CR group or the outpatient CR group. This separation was performed randomly by a computerized allocation system applying an algorithm at a proportion of 1:1. Investigators were not aware of the randomized matching sequence. The allocation was hidden until the completion of the baseline examinations in sequentially numbered, sealed, opaque envelopes. The patients and investigators were not blinded to the intervention.

Inclusion criteria of the study were: (a) age >18 years old, (b) diagnosed with CAD (angina pectoris, myocardial infarction in the last six months), (c) treated with cardiovascular revascularization (percutaneous coronary intervention or coronary artery bypass grafting), (d) prescribed recommended cardiovascular pharmacological treatment, (e) left ventricular ejection fraction> 45%, (f) low to moderate cardiovascular risk assessed by a cardiologist [27], (g) condition to perform cardiopulmonary exercise testing (CPET), (h) ownership and literacy with information and communication technologies (personal computer, telephone or mobile connection, and internet access).

Key exclusion criteria of the study were: (a) contraindication to CR, (b) potentially high cardiovascular risk, (c) implanted cardioverter–defibrillator or pacemaker, (d) residual coronary artery stenosis requiring revascularization, (e) orthopedic or neurological disability to exercise, (f) mental disadvantage making cooperation impossible.

All assessments were performed at the hospital clinic. Participants obtained a personal questionnaire (sex, age, diagnosis, and pharmacological treatment), a trial manual, and an educational booklet (healthy diet advice, cardiovascular risk factors management, and smoking cessation). All participants were asked to complete 36 sessions over 12 weeks. Telehealth CR participants were encouraged to visit two in-hospital individual supervised training sessions to familiarize themselves with telehealth devices (wearable sensor and web-based training diary).

### 2.3. Intervention

**Figure 2:**
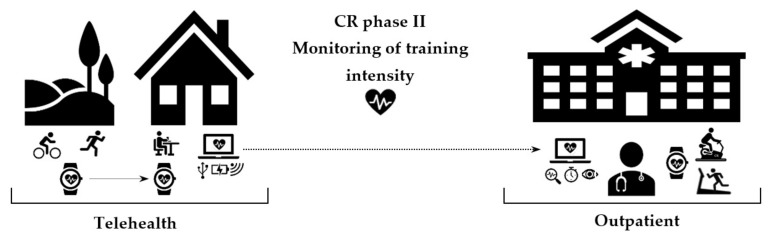
Scheme of monitoring training intensity.

The training intervention was prescribed on the principles of CR phase II (Figure 2), which consisted of regular physical exercise [28]. Both study groups included sixty minutes of continuous aerobic training, three times per week, guided using a wearable heart rate (HR) device (Polar 430M, Kempele, Finland).

A training HR zone was determined through baseline CPET assessment (70–80% HR reserve) and subsequently by the calculation [29]:

Training HR zone = [(peak HR − resting HR) × 0.7 to 0.8] + resting HR

The telehealth CR consisted of 36 training sessions three times a week. Moreover, once a week, each participant received a telephone consultation (guidance) based on the telemonitoring. Telephone guidance was predetermined for a specific day and time every week of intervention. Through the web-based training diary, the study physician supervised participants’ training sessions and analyzed the training data. The web-based training diary uses the internet to connect the wearable device to display recorded electronic health data (Figure 2). Telemonitored participants were contacted regularly every week. Based on the data in the training diary, the training adherence (intensity and duration) and the occurrence of adverse events related to the exercise were consulted. The telehealth CR consultations were approximately 10 to 20 min in duration. Participants allocated to the outpatient CR received training intervention under the direct supervision of a study physician and physical therapist at the hospital clinic. During training sessions at the outpatient clinic, participants received prescriptions on training intensity according to the training HR zone and numeral feedback from a wearable device. The clinical team recorded data of the training adherence in outpatient CR into a medical record.

### 2.4. Method of Data Collection

Study data were collected at baseline and throughout the 12-week training intervention period. Participants who completed at least 50% of the prescribed sessions (≥18 of 36) were included in the final analysis. A unique randomized selection (1 to 36) of six training sections was used for tabular expression of the comparison of training intensity results. Training intensity results (defined as a percentage of the HR reserve) were assessed and calculated from every participant training session using the following formula:

(mean training HR − resting HR/peak HR − resting HR) × 100

The formula expresses the parameters of resting HR (in a seated position) and peak HR that were assessed during the baseline CPET. The mean training HR was extracted from trial medical records and represented the mean HR value of the aerobic training phase of each session.

### 2.5. Cardiopulmonary Exercise Test

Determination of peak HR was achieved using a progressive incremental CPET on an Ergoselect 100 bicycle ergometer (Ergoline, Bitz, Germany) up to the symptom-limited maximum of each patient. CPET was completed in accordance with the European Society of Cardiology guidelines and the American Cardiology Association [30]. The role of CPET in clinical cardiology lies mainly in the means of diagnosis and prognosis as well as exercise training [31]. The peak and resting HR assessments with a standard 12-lead electrocardiogram, gas exchange, and blood pressure were continuously recorded and estimated through CPET. Blood pressure was measured manually with a CA-MI tonometer (Parma, Italy) every 2 min. A study physician supervised the testing in case of an adverse event or occurrence of complications. 

### 2.6. Statistical Analyses

The sample size calculation was based on predicted peak oxygen consumption improvement of 3.2 mL/kg/min with a standard deviation of 4.2 mL/kg/min reported in a previous study focused on the outpatient CR II phase [32]. Fifty-six participants were needed to achieve 80% statistical power with a level of significance alpha = 0.05. An estimated 10% drop-out was incorporated into the calculation. Thus, 28 participants had to be included in both study groups.

Study data were expressed using statistical descriptions, such as means and standard deviations for continuous variables and numerical variables. Continuous variables were compared between study groups using a *t*-test (“Student’s”) and numerical variables using the chi-squared test. A two-tailed Mann–Whitney U test was performed for collected data that had not been normally distributed. Training intensity was analyzed between study groups using a two-tailed Mann–Whitney test. The statistical significance level was set at *p* < 0.05 (two-tailed) for all differences between study groups. All study data were processed and analyzed in computerized statistical software Statistica 12 (TIBCO, Software Inc., Palo Alto, CA, USA).

## 3. Results

Table 1 summarizes participants’ descriptive characteristics, clinical data, and CPET outcomes for the experimental telehealth CR and the control outpatient CR group, with no statistically significant difference between groups. Reasons for dropouts are shown in Figure 1. The characteristics of the overall study sample were 85% male with a similar cardiovascular risk profile. Mean training HR was recorded at 117.6 ± 10.6 beats per minute in the telehealth CR group and at 115.9 ± 9.2 beats per minute in the outpatient CR group. The total training intensity adherence (defined as a percentage of the HR reserve) was similar: 74.8 ± 3.3% for telehealth CR compared to 75.3 ± 3.0% for outpatient CR (*p* = 0.63). Due to the low battery on the wearable HR device, a loss of 4.8% of training data in the telehealth CR group occurred.

**Figure 3:**
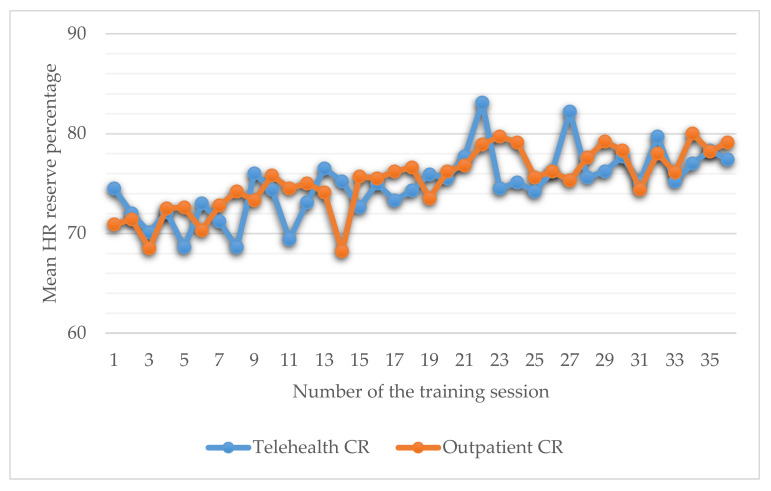
Graphic representation of training intensity between study groups.

No differences in training intensity were identified between study groups for each of the 36 rehabilitation sessions (Figure 3).

Data from randomly selected training sessions 2, 7, 16, 28, and 35 are shown in Table 2. The total time duration of the training phase at the prescribed HR was similar (*p* = 0.35) in both study groups: 54.0 ± 9.6 minutes (telehealth CR) and 56.4 ± 3.8 minutes (outpatient CR). The overall participation rate of all completed training sessions for telehealth CR was 33.3 ± 7.1 (92.6%) versus 30.5 ± 5.9 (85.5%) for outpatient CR (*p* = 0.24).

Out of the 1536 training sessions, there was no death case, and all severe adverse cases required medical admission throughout or within six hours after performing an exercise training session in study subjects in both research groups. Out of the 767 completed training sessions, telehealth CR reported four falls not requiring hospitalization (0.5%) versus no falls in outpatient CR. None of the participants experienced a new-onset cardiac event. There was one episode during the CPET when the participant was feeling weak. However, within minutes, there was a subsequent recovery with the help of medical staff, and no further treatment was required.

## 4. Discussion

This randomized control study recorded similar results in adherence to training intensity in the remotely monitored telehealth CR compared to the control outpatient CR II phase in the predominantly male population of CAD participants. The observed results encouraged the study’s research question and confirmed our assumption that the training intensity would not differ between the groups. In addition, there was similar time spent at the prescribed training intensity for both training groups (54.0 min and 56.4 min, respectively, *p* = 0.35). It should be emphasized that the frequency and intensity of training were objectively measured in all participants. This finding is critical to addressing concerns regarding achieving sufficient training adherence in telehealth CR. In our study based on vigorous training intensity (based on HR reserve percentage), adherence to participant training prescription was solidly maintained, providing an effective response to telehealth CR interventions. As demonstrated in the past, supervised training provides more targeted guidance with significant moderate- and high-intensity training [33]. 

As far as we know, our study is one of the first to address vigorous intensity in telehealth training where participants used wearable HR monitors to record training efforts. Kraal et al. [34] compared home-based and outpatient training at the 12-week intervention time based on moderate to vigorous intensity (prescribed according to 65–80% of HRmax) in participants with CAD. However, an objective comparison of exercise intensity measurements is lacking in this study. Keteyian et al. [35] recently compared the hybrid CR approach, combining the outpatient phase in the first half of the program with the remote approach. The study showed no significant difference in mean exercise intensity adherence during CR performed at home or in the community from what was observed during CR supervised at the clinic. The different intensity results compared to our study could be influenced by the heterogeneity of the study protocol. Another explanation may be that transformation of the training conditions from center-based to home-based could be complicated for patients to get used to, and, therefore, reduced training adherence may have occurred. On the other hand, in our study, patients became familiar with home-based training and remote monitoring from the beginning of the intervention, which could have a more beneficial effect on the approach. Furthermore, Avila et al. [36] demonstrated that home-based telerehabilitation exercise training leads to stable exercise capacity over one year following phase II cardiac rehabilitation.

Another approach to even higher gains is to use the high-intensity interval of exercise training [37]. Despite concerns about using high-intensity exercise intervals in the home environment [38], Aamot et al. [39] demonstrated a result confirming effectively performed CR concerning exercise intensity and exercise attendance compared to supervised exercise in the clinical center. Furthermore, based on the above, is the improvement in aerobic capacity levels clinically significant. As was demonstrated, an increase of 1 mL/kg/min in oxygen consumption is associated with an improvement in prognosis (approximately two years) and a reduced risk of further cardiovascular events and mortality [40,41].

Telehealth CR has been associated with increased safety concerns and the risk of adverse events, particularly in the older CAD participant population [42]. The first meta-analysis on the effectiveness of cardiac telerehabilitation found no differences between the effectiveness of this form versus traditional rehabilitation in low-to-moderate-risk CAD patients [43]. Moreover, according to reports by Buys et al. covering a group of over 300 patients (mean age 61.7 ± 14.5) during phase II of CR, the patients presented a great interest in new technologies used in the rehabilitation process. It was shown that 91% of respondents used the internet regularly, 97% had a mobile phone, 64% used smartphone applications, and 79% previously used computer games that generated physical effort [44]. A recently published review paper of telehealth CR studies on 545 participants [45] showed no occurrence of causes of death or serious complications associated with training intervention, which is consistent with our result. No statistically significant major events were recorded, only four falls in the telehealth group. However, the challenge of typical clinical concerns, such as arrhythmias, hemodynamic changes, dizziness, or falls, has not been mitigated by any research to date [46]. A future trend that predicts an increase in CAD population and total age (presumption of fragility and comorbidity) may pose an additional risk [47]. Consequently, addressing the clinical safety of telehealth CR requires greater emphasis and further investigation.

Thus, this study shows that telehealth CR with vigorous intensity can be effectively performed in CAD patients if baseline symptom assessment and limited CPET are performed. Further research is needed to optimize the quality of training provided in telehealth CR and, in particular, to identify or develop additional training modalities that can improve training adherence (e.g., strength training or yoga) [48,49].

Finally, our research provides data that address training adherence in telehealth CR interventions, where there is usually a concern that training in the home environment is followed at lower intensities than in outpatient CR. We expect that our research will be able to optimize the current dynamic development in alternative approaches in CR.

### Limitations

The main limitation observed in our study is that this is the one-center result with a population of participants from the Moravian region of the Czech Republic. Therefore, the result may not be generalizable to other CR participants from other regions. A clinical study of multiple centers should be conducted to resolve the above limitation. Secondly, our study included only a small percentage of women. Therefore, the results are difficult to adapt for this population group. Thirdly, although the training intensity was accurately determined through a wearable HF monitor, several patients experienced problems with an insufficient charge of the device. As a result, some patients discontinued prescribed training early, which may have led to lower data reliability. Finally, there is a need to acknowledge that our telehealth CR method may not be available and feasible for some CR centers, as the language version of the web application, staff readiness, or limited access to technology can be limiting. However, due to the SARS-CoV-2 pandemic, clinical medicine services are now better prepared and equipped for the needs of remote and/or virtual CR programs [50].

## 5. Conclusions

This research demonstrated that the telehealth CR model utilized via remote telemonitoring is similar in terms of training intensities to the conventional outpatient CR in CAD patients with low to moderate cardiovascular risk. Thus, the result supports telehealth implementation in rehabilitation practice as an alternative approach. It seems reasonable in the era of current pandemic restrictions to consider a wider use of telerehabilitation in phase II CR patients as a low-cost approach to provide comparable results on a large scale.

## Figures and Tables

**Table 1:** Baseline demographic and clinical characteristics of the participants.

Participant Characteristics	Telehealth CR (*n* = 23)	Outpatient CR (*n* = 25)	*p*-Value
Age (years)	56.1 ± 6.8	57.2 ± 7.5	0.62
Sex male, n (%)	19 (79)	22 (84)	0.60
BMI (%)	28.1 ± 3.5	28.7 ± 4.3	0.54
LVEF (%)	59.9 ± 5.9	58.2 ± 5.8	0.36
Diagnosis			0.19
AP, n (%)	5 (22)	2 (8)	
AMI, n (%)	18 (78)	23 (92)	
Intervention			0.61
PCI, n (%)	19 (83)	22 (88)	
CABG, n (%)	4 (17)	3 (12)	
Cardiovascular risk factors			
Hypertension, n (%)	13 (57)	15 (60)	0.81
Diabetes, n (%)	6 (26)	5 (20)	0.62
Dyslipidemia, n (%)	10 (44)	14 (56)	0.39
Smoking, n (%)	14 (61)	12 (48)	0.38
Family history, n (%)	10 (44)	8 (32)	0.42
Waist circumflex (cm)	102.7 ± 10.4	103.1 ± 12.9	0.82
Baseline CPET			
Peak heart rate, bpm	138.8 ± 17.5	137.9 ± 16.9	0.87
peak VO_2_, mL/kg/min	23.7 ± 4.0	23.1 ± 3.0	0.58
peak RER, unit	1.2 ± 0.1	1.2 ± 0.0	0.74

AP, angina pectoris; AMI, acute myocardial infarction; BMI, body mass index; CABG, coronary artery bypass graft; CPET, cardiopulmonary exercise test; CR, cardiac rehabilitation; LVEF, left ventricular ejection fraction; PCI, percutaneous coronary intervention; RER, respiratory exchange ratio, VO_2_, oxygen consumption.

**Table 2:** Results of the training intensity in telehealth and outpatient CR.

Training Session Number	Telehealth CR ^1^		Outpatient CR ^1^		*p*-Value
Session 2	*n* = 22	72.0 ± 3.1%	*n* = 22	71.4 ± 2.8%	0.77
Session 7	*n* = 21	71.2 ± 2.9%	*n* = 20	72.8 ± 3.5%	0.76
Session 16	*n* = 17	75.0 ± 4.6%	*n* = 23	75.5 ± 3.7%	0.52
Session 28	*n* = 23	75.6 ± 4.2%	*n* = 23	77.6 ± 3.3%	0.41
Session 35	*n* = 19	75.6 ± 3.4%	*n* = 24	77.2 ± 3.4%	0.35

^1^—Mean training intensity expressed as heart rate reserve percentage; data are described as mean ± standard deviation.

## Data Availability

The data presented in this study are available on request from the corresponding author. The data are not publicly available.
